# Targeting the innate repair receptor axis *via* erythropoietin or pyroglutamate helix B surface peptide attenuates hemolytic-uremic syndrome in mice

**DOI:** 10.3389/fimmu.2022.1010882

**Published:** 2022-09-23

**Authors:** Sophie Dennhardt, Wiebke Pirschel, Bianka Wissuwa, Diana Imhof, Christoph Daniel, Jan T. Kielstein, Isabel Hennig-Pauka, Kerstin Amann, Florian Gunzer, Sina M. Coldewey

**Affiliations:** ^1^ Department of Anesthesiology and Intensive Care Medicine, Jena University Hospital, Jena, Germany; ^2^ Septomics Research Center, Jena University Hospital, Jena, Germany; ^3^ Center for Sepsis Control and Care, Jena University Hospital, Jena, Germany; ^4^ Pharmaceutical Biochemistry and Bioanalytics, Pharmaceutical Institute, University of Bonn, Bonn, Germany; ^5^ Department of Nephropathology, Friedrich-Alexander University (FAU) Erlangen-Nürnberg, Erlangen, Germany; ^6^ Medical Clinic V, Nephrology | Rheumatology | Blood Purification, Academic Teaching Hospital Braunschweig, Braunschweig, Germany; ^7^ Field Station for Epidemiology, University of Veterinary Medicine Hannover, Bakum, Germany; ^8^ Department of Hospital Infection Control, University Hospital Carl Gustav Carus, TU Dresden, Dresden, Germany

**Keywords:** erythropoietin, hemolytic-uremic syndrome, shiga toxin, mice, pyroglutamate helix B surface peptide, microangiopathic hemolytic anemia, thrombotic microangiopathy

## Abstract

Hemolytic-uremic syndrome (HUS) can occur as a systemic complication of infections with Shiga toxin (Stx)-producing *Escherichia coli* and is characterized by microangiopathic hemolytic anemia and acute kidney injury. Hitherto, therapy has been limited to organ-supportive strategies. Erythropoietin (EPO) stimulates erythropoiesis and is approved for the treatment of certain forms of anemia, but not for HUS-associated hemolytic anemia. EPO and its non-hematopoietic analog pyroglutamate helix B surface peptide (pHBSP) have been shown to mediate tissue protection *via* an innate repair receptor (IRR) that is pharmacologically distinct from the erythropoiesis-mediating receptor (EPO-R). Here, we investigated the changes in endogenous EPO levels in patients with HUS and in piglets and mice subjected to preclinical HUS models. We found that endogenous EPO was elevated in plasma of humans, piglets, and mice with HUS, regardless of species and degree of anemia, suggesting that EPO signaling plays a role in HUS pathology. Therefore, we aimed to examine the therapeutic potential of EPO and pHBSP in mice with Stx-induced HUS. Administration of EPO or pHBSP improved 7-day survival and attenuated renal oxidative stress but did not significantly reduce renal dysfunction and injury in the employed model. pHBSP, but not EPO, attenuated renal nitrosative stress and reduced tubular dedifferentiation. In conclusion, targeting the EPO-R/IRR axis reduced mortality and renal oxidative stress in murine HUS without occurrence of thromboembolic complications or other adverse side effects. We therefore suggest that repurposing EPO for the treatment of patients with hemolytic anemia in HUS should be systematically investigated in future clinical trials.

## Introduction

Hemolytic-uremic syndrome (HUS) belongs to the group of thrombotic microangiopathies and includes atypical and typical HUS. The latter accounts for approximately 90% of HUS cases and is a life-threatening systemic complication of infections with certain bacterial pathogens, most commonly Shiga toxin (Stx)-producing *Escherichia coli* (STEC) (1).

STEC-infections can cause diarrhea or hemorrhagic colitis with bloody diarrhea. STEC-HUS typically presents with microangiopathic hemolytic anemia, thrombocytopenia, acute kidney injury (AKI) and other organ dysfunctions (1). One-third of patients with STEC-HUS also develop long-term renal and up to 4%, long-term neurological sequelae (2). Renal oxidative stress was shown to be an important factor in HUS pathogenesis in pediatric HUS (3, 4). Its pathophysiological relevance was also demonstrated in a murine model of Stx2-induced HUS (5).

To date, organ supportive therapy in the intensive care unit (ICU), including hemodialysis, fluid resuscitation as well erythrocyte transfusion if indicated, has been the standard of care in patients with STEC-HUS (6). In the absence of targeted therapeutic options, there is a medical need to further investigate molecular therapeutic approaches for the treatment of this life-threatening systemic syndrome.

Due to the low incidence – of e. g. 0.07 per 100,000 persons/year for Germany (7) up to 0.67 per 100,000 persons/year for Argentina (8) – conducting prospective randomized clinical trials has proven difficult. To provide tools for preclinical studies, we have recently introduced and characterized several animal models for STEC-HUS, including an infection model employing the Northern German outbreak strain O104:H4 of 2011 and the well-characterized outbreak strain O157:H7 86-24 in gnotobiotic piglets (9), as well as a clinically relevant mouse model (10–12) reflecting most aspects of human pathology by repeatedly exposing animals to low doses of Stx isolated from an EHEC O157:H7 86-24. All previously described small animal models of STEC-HUS mimic well the critical illness, renal failure and microangiopathy seen in humans, while being limited in their modeling of hemolytic anemia, which is oftentimes masked by hemoconcentration secondary to fluid deficiency (10, 13, 14).

Erythropoietin (EPO), a pleiotropic hormone that has been shown to exert tissue-protective effects *via* the innate repair receptor (IRR) independent of its hematopoietic properties *via* the EPO receptor (EPO-R) homodimer (15, 16), appears to be a promising candidate to be further evaluated in HUS. Preclinical trials usually use one magnitude higher doses of EPO compared with clinical trials (1000-5000 IU/kg versus 300 IU/kg) (17). This could explain why preclinical tissue protection following EPO administration could often not be translated into the clinical setting. Furthermore, beside its potential beneficial effects, the thrombogenic effect of this erythropoiesis-stimulating hormone was critically discussed in the context of clinical studies (18, 19), as tissue-protective effects require high levels of EPO and administration of high EPO doses can increase the risk of thrombosis (16) and hypertension (17). For this reason, EPO-derived non-hematopoietic small peptide activators of the IRR, such as the pyroglutamate helix B surface peptide (pHBSP), have been developed (15). Preclinically, pHBSP has been shown to convey tissue protection in numerous disease models (20), such as nephroprotection in ischemia-reperfusion models of AKI (21, 22). Furthermore, pHBSP has been successfully employed in phase II studies in patients with diabetes or sarcoidosis and neuropathic pain (23, 24).

HUS can be considered as a specific phenotype of sepsis, as it represents a combination of infection and organ dysfunction. We previously reported that EPO attenuated AKI (25) and cardiac dysfunction (26) in mouse models of endotoxemia and cecal ligation and puncture-induced sepsis. There is no clinical evidence on the beneficial or harmful effects of EPO in patients with sepsis and there is only little evidence regarding the beneficial or harmful effects of EPO in ICU patients in general (27, 28). To our knowledge, the tissue-protective effects of EPO or pHBSP in HUS have not been investigated, yet. One trial that aimed to investigate the effects of EPO on the microcirculation of patients with severe sepsis was discontinued due to lack of recruitment (NCT01087450). We did not find the results of a study that examined the immunomodulatory effects of activated protein C and/or EPO in sepsis (NCT00229034). A third study examining the effects of EPO on renal function in critically ill patients with and without multiorgan failure appears to be not completed (EudraCT number: 2008-003733-24). Altogether, there is no evidence from randomized controlled trials regarding the effects of EPO administration in sepsis and HUS. Despite this lack of evidence, it is however conceivable, that in the presence of severe hemolytic anemia in patients with HUS, the stimulation of hematopoiesis by EPO could be beneficial by reducing the need for allogenic erythrocyte transfusion, which carry non-negligible risks (29, 30).

Anemia is usually accompanied by a regulatory increase in endogenous EPO production due to hypoxia-mediated feedback mechanisms. In patients with iron deficiency or hemolytic anemia, serum EPO levels exceeding 2000 mU/ml (two orders of magnitude above normal range) have been reported (31). In contrast, renal anemia is often accompanied by insufficient EPO production due to direct damage to EPO-producing cells in the kidney (32) or inhibition of EPO production by various cytokines (33). Already in the early 1990’s, it was hypothesized that anemias associated with low serum EPO levels might respond to treatment with recombinant EPO (34).

Currently, EPO can be considered in the treatment of anemia associated with chemotherapy (35) and chronic kidney disease (36). The current national guideline for the treatment of HUS in pediatric patients states that EPO can be considered in the treatment of HUS-associated hemolytic anemia as expert consensus without citing evidence (37). So far, no adverse effects have been observed in children receiving EPO for the treatment of hemolytic anemias (38–41). However, a reduction of red blood cell transfusion could not be shown in two small studies (38, 39). Hitherto, there are no sufficiently powered clinical studies in this context. Even a neutral effect of EPO in terms of nephroprotection in the absence of side effects might positively impact the therapy of HUS-associated hemolytic anemia and save the need for red blood cell transfusions.

In view of the above, we consider it necessary and promising to assess the role of EPO in this orphan disease which comprises a specific form of septic organ failure. The first objective of this study is to further elucidate the role of endogenous EPO and its potential clinical relevance in HUS by measuring endogenous EPO levels in different species: human patients (infection with EHEC O104:H4, 2011 German outbreak), gnotobiotic piglets (subjected to EHEC O104:H4 or EHEC O157:H7 86-24) and mice (subjected to Stx2 derived from EHEC O157:H7 86-24). The second objective of this study is to examine the effect of EPO and the non-hematopoietic peptide pHBSP in a murine model of HUS by measuring surrogate parameters of kidney injury and dysfunction, intrarenal barrier integrity, microangiopathy, oxidative and nitrosative stress and metabolome. Hereby, we aim to provide preclinical evidence to assess whether treatment of HUS with EPO or pHBSP should be considered and further investigated, particularly for HUS-associated hemolytic anemia.

## Material and methods

### Study design

Plasma samples of patients with STEC-HUS (n = 27, median age 47, 24 female) were provided by the German STEC-HUS Registry (42). These samples were taken within 10 d after admission to the hospital (HUS acute stage) and at the day of the last plasmapheresis (HUS pre-discharge). We analyzed the subcohort of 7 patients of which samples were available from day 1 to day 3 after hospital admission and within 3 d before hospital discharge. Age- and sex-matched samples of healthy controls (n = 21, median age 63.5, 15 female) were provided by Jena University Hospital [ICROS study (43)]. Ethic approval was obtained by the primary investigators from the Ethics Committee of Hannover Medical School (1123-2011) and of the Friedrich Schiller University Jena (5276-09/17). Participants provided written informed consent prior to inclusion in the respective studies.

Samples from gnotobiotic piglets (9) were collected 4 to 6 days after STEC-infection or mock infection (sham). Experimental procedures in gnotobiotic piglets were approved by the local permitting authorities in the Lower Saxony State Office for Consumer Protection and Food Safety and in accordance with the requirements of the national animal welfare law (Approval Number: 33.9-42502-04-13/1149) in accordance with the German legislation following the guidelines of FELASA and ARRIVE (9).

All procedures performed in mice were approved by the regional animal welfare committee and the Thuringian State Office for Consumer Protection (registration number 02-058/14) and performed in accordance with the German legislation. HUS-like disease in mice was induced by repetitive doses of Stx2 as described previously (10). Briefly, wild-type C57BL/6J mice aged 10-16 weeks weighing 20-30 g were randomly assigned to one of four groups (sham n = 16, Stx+vehicle n = 26, Stx+EPO n = 22, or Stx+pHBSP n = 21) and received 3x25 ng/kg body weight (BW) Stx or 0.9% NaCl i.v. on days 0, 3, and 6. EPO (1000 IU/kg BW) was applied s.c. 1 h after initial Stx injection. Due to its short plasma half-life, pHBSP (30 µg/kg BW) was applied s.c. every 24 h starting 1 h after initial Stx injection. Vehicle (Ringer’s solution) was applied s.c. starting 1 h after initial Stx injection. Mice received 3x800 µl Ringer’s solution each day for volume replacement. In compliance with ethical regulations, survival was monitored for 7 days using humane endpoints (mice were euthanized when reaching a high-grade disease state, **Figure 3**). Disease progression was monitored by weight loss and activity-based HUS score (ranging from 1-normally active, 2-active with slight restrictions, 3-active with clear intermissions, 4-slowed, 5-lethargic, 6-moribund, to 7-dead) as described previously (10). Animals were exsanguinated in deep ketamine/xylazine anesthesia (10). Renal tissue, blood and plasma samples were collected on day 7. An additional experiment with a comparable survival rate including sham and Stx+vehicle mice was performed to compensate for the lower survival rate in the Stx+vehicle group and to increase the statistical power in the analysis of day-7-samples for plasma urea and creatinine and all histological [Schiff’s periodic acid (PAS), acid fuchsin orange G (SFOG)] and immunohistochemistry (IHC) stainings (kidney-injury molecule 1 (KIM-1), cluster of differentiation 31 (CD31), E-cadherin, glycoprotein 1b (GP1b), nitrotyrosine and NADPH oxygenase 1 (NOX-1), **Figures 4**–**7**). Plasma EPO levels (**Figure 2B**) and plasma metabolome (**Figure 8**) were measured in an independent experimental setup with comparable survival rates since availability of plasma per animal was limited.

### Compounds

Stx purification was performed as described previously (10). Human recombinant EPO (Epoeitin beta, Hoffmann-La Roche) was diluted to 1000 U/kg BW in Ringer’s solution (vehicle). A standard N-(9-fluorenyl)methoxycarbonyl protocol for automated solid-phase peptide synthesis was implemented for pHBSP synthesis (**Supplementary Methods**). pHBSP was diluted to 30 µg/kg BW in Ringer’s solution (vehicle).

### Blood and plasma sample analysis

Human (42), piglet (9) and murine (10) blood samples were taken as described elsewhere. Hematology and analysis of laboratory chemistry parameters in murine samples were performed as recently described (10). Briefly, blood counts were analyzed using impedance technology implemented in the pocH100iv system (Sysmex, Kobe, Japan). Laboratory parameters were analyzed by an Architect™ c16200/ci8200 automated clinical chemistry system (Abbott Diagnostics, Abbott. Park, USA). This system uses the Jaffè method for plasma creatinine measurements and the urease/nicotinamide adenine dinucleotide hydrogen method for plasma urea measurements. The following parameters were measured using commercially available ELISA kits: serum or plasma EPO and murine plasma neutrophil-gelatinase-associated lipocalin (NGAL, **Supplementary Table S1**).

### Tissue preparation, histopathology and IHC staining

Processing of kidney samples, histopathological analysis and IHC stainings were performed as described previously (10). SFOG and thrombocyte (GP1b) staining was performed as described recently (11). Until antigen demasking, sections for E-cadherin, NOX-1 and nitrotyrosine staining were treated similarly. The Vector M.O.M. Immunodetection kit (Vector Laboratories) was used for E-cadherin staining. Blocking of nitrotyrosine staining was performed in normal goat serum. IHC sections were washed with Tris(hydroxymethyl)aminomethan (TRIS) buffer during staining [50 mM TRIS, 300 mM NaCl, pH was adjusted to 7.6 with HCl (all Carl Roth); 0.04% Tween^®^20 (Sigma Aldrich)], incubated with primary antibodies overnight at 4°C in appropriate dilutions (**Supplementary Table S2**) and subsequently incubated for 30 min with secondary antibodies (**Supplementary Table S3**). NOX-1 sections were incubated in primary antibody directly after antigen retrieval. Nitrotyrosine sections were stained using the CSA kit and rabbit Link (Dako).

### Histology and IHC quantification

Quantification and scoring of histopathological and IHC stainings were performed as described previously (10). For quantification of renal E-cadherin expression, all cut and completely positively stained tubules (with entirely visible lumen, excluding those with spotty staining) were counted in 12 adjacent cortical fields [one cortical field = region of interest (ROI)] per section in 400x magnification. Nitrotyrosine staining was quantified by superimposing a 10x10 grid **(**area of 0.0977 mm²) over each field and counting positively stained fields in 20 adjacent cortical fields per section in 400x magnification (blinded). NOX-1 staining was analyzed using a scoring system (0 to 3; 0: < 25%, 1: 25-50%, 2: 50-75%, 3: > 75% positive staining per field, 12 fields/section, magnification 200x, blinded). Quantification of SFOG and thrombocyte staining was performed as described recently (11).

### Targeted metabolomics and statistical analysis

Targeted metabolomics from heparinized plasma was performed at Biocrates Life Sciences by mass spectrometry using an MxP^®^ Quant 500 kit. Data analysis was performed using R version 3.4.4 (R Core Team) as follows: Readings below detection level were set to half of detection level for each analyte separately. Metabolome data was then log 2 transformed without any further normalization. Z scores were calculated using mean and standard deviation of all samples. Trends along the four sample groups were tested using linear regression models where sample group assignment was used as the independent variable and each analyte as the dependent variable in a separate model. Prior to linear regression, the independent variable sample group assignment was transformed to a pseudo-continuous variable where all samples of group “sham” were set to 0, samples of group “Stx+pHBSP” were set to 1/3, samples of group “Stx+EPO” were set to 2/3 and samples of group “Stx+vehicle” were set to 1. Applying this transformation, the linear regression models test whether there is an increasing or decreasing trend along the four groups in the assigned order. P-values for all models were Benjamini-Hochberg adjusted (44). Analytes with adjusted P-values below 0.05 and an absolute effect of one or bigger were considered significantly changing along the four sample groups.

### Statistics

Data are depicted as mean ± SD for n observations. GraphPad Prism 7.05 (GraphPad Software) was used for data analysis applying Student’s t-test and Wilcoxon signed rank test for comparisons between 2 groups. One-way ANOVA with Holm-Sidak *post hoc* test (parametric data) or Kruskal-Wallis test with Dunn’s *post hoc* test (nonparametric data) were used for comparisons between more than 2 groups. Survival was depicted as Kaplan-Meier curve and analyzed by Mantel-Cox test. Association between EPO levels in patients with STEC-HUS and hematological and laboratory parameters and piglet samples was performed with GraphPad Prism 7.05 implementing non-parametric Spearman’s rank correlation coefficients. A P-value < 0.05 was considered significant. Mean ± SD and P-values for all analyses are given in **Supplementary Table S4**.

## Results

### Elevated EPO levels in humans suffering from HUS

**Figure 1:**
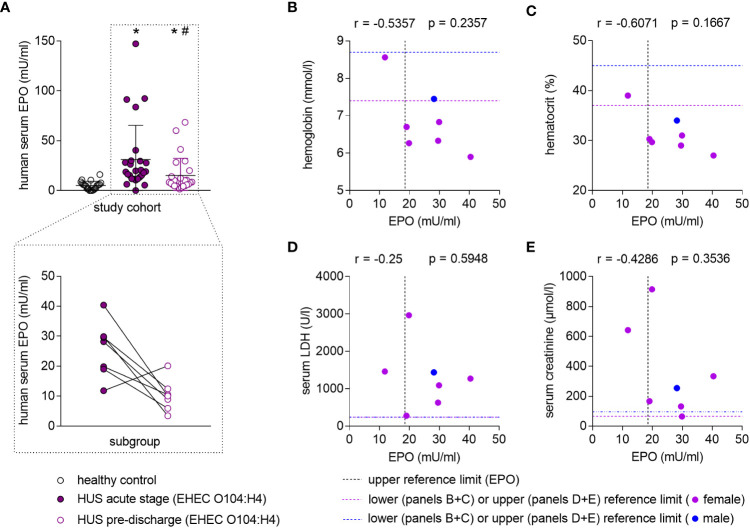
Endogenous EPO levels in patients with STEC-HUS and their correlation with anemia and kidney damage. **(A)** Upper panel: EPO serum levels in healthy controls (n = 20) and HUS patients (n = 26) within 10 d after admission to the hospital (HUS acute stage) and the last plasmapheresis (HUS pre-discharge) determined by ELISA. Data are expressed as scatter dot plot with mean ± SD. *P < 0.05 vs. healthy control (Mann-Whitney U-test), ^#^P < 0.05 vs. HUS acute stage (Wilcoxon signed-rank test). Lower panel: Pairwise comparison of EPO serum levels of a subcohort of patients (n = 7) in which blood samples were available from day 1 to 3 after hospital admission and within 3 days before hospital discharge. Data are expressed as scatter dot plot with connecting line between respective measurements. **(B-E)** Correlation analysis implementing Spearman’s correlation coefficient (r) for correlations of endogenous EPO levels with **(B)** hemoglobin (n = 7), **(C)** hematocrit (n = 7), **(D)** lactate dehydrogenase (LDH, n = 7) and **(E)** creatinine (n = 7) in the subcohort. The upper reference limit for EPO in health are indicated by dashed vertical lines. Gender-specific lower or upper reference limits for the respective parameters of anemia and kidney damage are indicated by dashed horizontal lines.

In patients with STEC-HUS, EPO serum levels were elevated in the acute stage of disease compared with healthy controls (**Figure 1A**, upper panel, **Supplementary Tables S5, S6** for individual values). Pre-discharge, EPO levels decreased compared to the acute phase, yet continued to be higher than in healthy controls. In a subgroup, in which blood samples were consequently taken within 3 days after admission and 3 days before discharge from the hospital, a reduction of serum EPO levels was observed in 6 out of 7 HUS patients (**Figure 1A**, lower panel). Several hematological, laboratory and clinical parameters at hospital admission are listed in **Table 1** and EPO values were correlated with these parameters. Anemia was present in 6 out of 7 patients (**Figures 1B, C**) and there was a trend towards a negative correlation between endogenous EPO levels and hemoglobin (Hb; r = -0.5357, P = 0.2357) or hematocrit (r = -0.6071, P = 0.1667) at hospital admission. No correlation was observed between endogenous EPO levels and lactate dehydrogenase (LDH) or creatinine at hospital admission (**Figures 1D, E**), although intriguingly, the lowest endogenous EPO levels were observed in patients with pronounced kidney dysfunction (**Figure 1E**).

**Table 1:** Hematological, laboratory and renal function parameters in the subgroup of patients with STEC-HUS assessed at hospital admission.

Patient no.	Sex	Age	EPO (T1, mU/ml)	Hb (g/dl)	Hct (%)	LDH (U/l)	Crea (mg/dl)	Dialysis	Plasmapheresis(number)	RBC
5	m	26	28.194	12.0	34.0	1437	2.88	no	yes (6)	no
9	f	74	19.856	10.1	29.7	2966	10.35	yes	yes (4)	n/a
11	f	29	40.382	9.5	27.0	1273	3.78	yes	yes (6)	yes
12	f	39	29.546	10.2	29.0	627	1.49	no	no	no
13	f	44	19.034	10.8	30.3	279	1.89	no	yes (4)	yes
15	f	50	29.914	11.0	31.0	1091	0.73	yes	yes (7)	yes
25	f	73	11.820	13.8	39.0	1460	7.26	yes	yes (5)	yes

Hb, hemoglobin; Hct, hematocrit; LDH, lactate dehydrogenase; Crea, creatinine; RBC, red blood cell transfusion.

While anemia appears to be a major driver of increased EPO secretion in patients with STEC-HUS, we wondered whether other mechanisms might also play a role. Considering that – at least in our limited subcohort-endogenous EPO was lowest in those patients with pronounced kidney dysfunction and injury, we hypothesize that these patients might profit from therapeutic EPO administration.

### Elevated EPO levels in animal models of HUS

**Figure 2:**
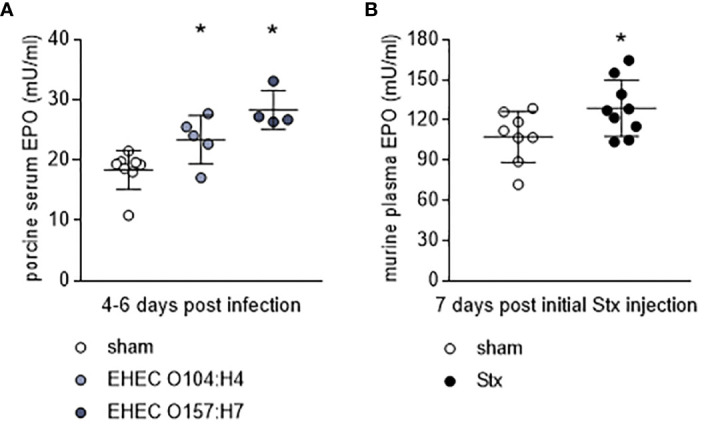
Endogenous EPO levels in animal models of HUS. **(A)** EPO serum levels in gnotobiotic piglets that were either mock-infected (sham) or infected with EHEC O157:H7 or EHEC O104:H4 determined by ELISA. Samples were taken 4-6 d after infection (sham n = 8, EHEC O104:H4 n = 5, EHEC O157:H7 n = 4). Data are expressed as scatter dot plot with mean ± SD. *P < 0.05 vs. sham (one-way ANOVA, Holm-Sidak’s multiple comparison test). **(B)** EPO plasma levels in mice with HUS on day 7 of experiment (sham n = 8, Stx n = 10) determined by ELISA. Data are expressed as scatter dot plot with mean ± SD, *P < 0.05 vs. sham (unpaired t-test).

To further assess the role of EPO in HUS, we measured endogenous EPO levels in two well-characterized animal models of this condition. In gnotobiotic piglets with STEC-HUS, EPO levels were elevated in EHEC O104:H4- and EHEC O157:H7-infected piglets compared with sham piglets (**Figure 2A**). STEC-infected gnotobiotic piglets did not display clear signs of anemia or kidney injury compared with mock-infected sham animals (**Table 2**). No correlations were observed between EPO-levels and hematological parameters (**Supplementary Table S7**). In O157:H7-infected piglets, although LDH was comparably low, there was a trend towards negative correlation with EPO levels (**Supplementary Table S7**). In C57BL/6 mice subjected to Stx, slight hemoconcentration was observed (**Supplementary Table 3**, significant increase in Hb in Stx+vehicle compared with sham mice). Despite the absence of anemia in Stx+vehicle mice, EPO plasma levels were elevated compared with sham mice (**Figure 2B**). There was no correlation between plasma EPO levels and plasma NGAL as surrogate parameter for kidney damage in mice (**Supplementary Figure S1**).

**Table 2:** Hematological, laboratory and renal function parameters in STEC-infected piglets and mock-infected controls (sham) assessed 4-6 days after infection.

-	ID	EPO (U/l)	Hb (g/l)	Hct (L/l)	LDH (U/l)	Crea(µmol/l)
sham	16	17.983	94.9	0.25	702	59
	32	19.211	95.86	0.27	1105	63
	33	10.827	79.3	0.24	901	57
	44	19.726	124.02	0.19	1445	67
	46	19.331	60.35	0.19	884	74
	47	21.491	69.92	0.21	924	73
	48	18.587	54.46	0.17	1145	60
	49	19.578	60.72	0.18	808	70
O104:H4	18	17.048	69.6	0.21	909	54
	19	25.473	97.5	0.27	997	59
	36	27.684	78.2	0.22	1767	73
	42	24.029	116.66	0.24	977	53
	43	22.659	96.42	0.29	1761	63
O157:H7	20	33.098	78.8	0.23	538	61
	21	26.325	97.9	0.26	804	52
	34	26.667	65.14	0.33	740	65
	35	27.173	*n.d.*	*n.d.*	680	72

Hb and Hct could not be assessed in one piglet of the O157:H7 group (n.d.). Hb, hemoglobin; Hct, hematocrit; LDH, lactate dehydrogenase; Crea, creatinine.

As anemia appears not to be the sole driver of increased EPO levels during HUS and patients with pronounced kidney damage showed the lowest EPO levels, we next wanted to analyze the therapeutic potential of EPO and the non-hematopoietic EPO derivative pHBSP in a murine model of HUS.

### Effect of EPO and pHBSP treatment on survival and clinical presentation of mice with HUS

**Figure 3:**
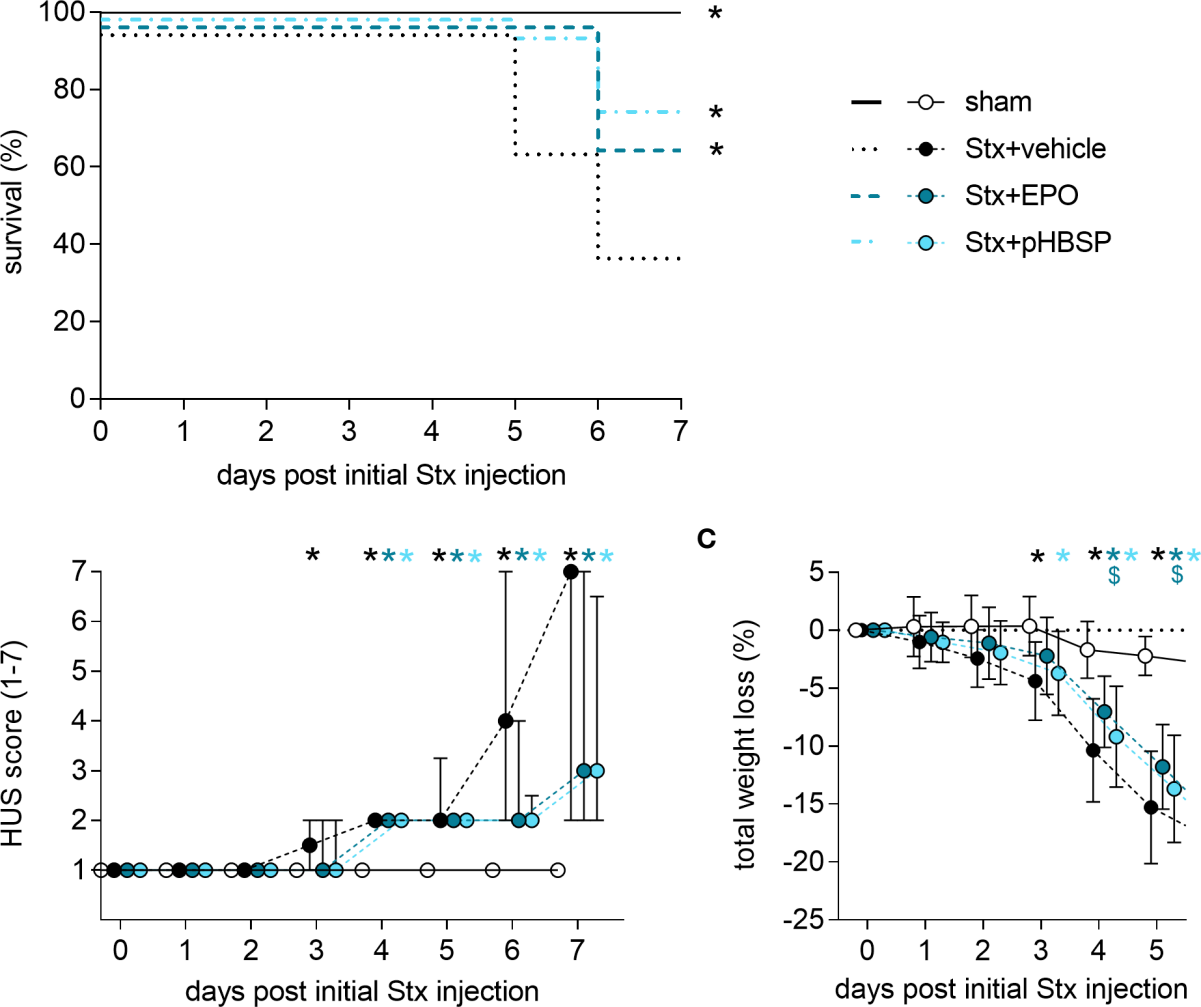
Effect of EPO and pHBSP treatment on survival and clinical presentation of mice with HUS. **(A-C)** Mice received either Stx or vehicle intravenously to induce experimental HUS. Mice with HUS were treated with vehicle (Ringer’s solution 1 h post HUS induction s.c.), EPO (1 h post HUS induction s.c., 1000 IU/kg BW once) or pHBSP (1 h post HUS induction s.c., 30 µg/kg BW every 24 h; sham n = 15, Stx+vehicle n = 26, Stx+EPO n = 22, Stx+pHBSP n = 21). **(A)** Kaplan-Meier 7-day survival curves of sham mice, Stx-challenged mice treated with vehicle, Stx-challenged mice treated with EPO and Stx-challenged mice treated with pHBSP with humane endpoints. *P < 0.05 vs. Stx+vehicle (log-rank Mantel-Cox test). **(B)** Activity-based HUS score (ranging from 1-normally active, 2-active with slight restrictions, 3-active with clear intermissions, 4-slowed, 5-lethargic, 6-moribund, to 7-dead) was assessed three times daily and is depicted every 24 h until day 7 starting with first Stx injection. Data are expressed as median ± interquartile range. *P < 0.05 vs. sham (Kruskal-Wallis test, Dunn’s multiple comparison test at every single time point). **(C)** Total weight loss referred to day 0 was assessed every 24 h and is depicted until day 5. Data are expressed as mean ± SD. *P < 0.05 sham vs. the respective color-coded group, ^$^P < 0.05 vs. Stx+vehicle (two-way ANOVA, Holm-Sidak’s multiple comparison test).

7-day survival was significantly increased in Stx+EPO (68.2%) and Stx+pHBSP mice (76.2%) compared with Stx+vehicle mice (42.3%; **Figure 3A**). Consistently, disease progression was reduced on days 6 and 7 in these treatment groups (**Figure 3B**). All surviving Stx-challenged animals lost up to 20% weight until the end of the experiment. Statistical comparison of weight loss was performed until the first animals had to be euthanized at day 5 (**Figure 3C**). Weight loss in Stx+EPO mice was less pronounced compared with Stx+vehicle mice on days 4 and 5 (**Figure 3C**). While neurological involvement assessed by hind limb clasping reflex frequently occurred in Stx+vehicle mice (approx. 39%, P = 0.0067 vs. sham, **Table 3**), it was less common in Stx+EPO (approx. 27%; **Table 3**) and Stx+pHBSP mice (approx. 24%; **Table 3**). No differences were observed in plasma alanine aminotransferase (ALAT) and aspartate aminotransferase (ASAT), while Hb and/or hematocrit as indicators of hemoconcentration were increased in all Stx-challenged mice (**Table 3**). Compared with sham mice, Hb was elevated in Stx+EPO mice, while it was not in Stx+pHBSP mice. However, there was no significant difference between Stx+EPO mice and Stx-vehicle mice. The effects of EPO-treatment on Hb might be masked by the slight hemoconcentration observed in all Stx-challenged groups.

**Table 3:** Hematological and laboratory parameters of mice with HUS and effects of EPO and pHBSP treatment.

		sham	Stx+vehicle	Stx+EPO	Stx+pHBSP	P-values
**clinical appearance**	neurological symptoms	0/15 (0%)	10/26 (39%)	6/22 (27%)	5/21 (24%)	sham vs. Stx+vehicle: P = 0.0067
**hematology**	Hct (%)	34.8 ± 1.6	38.7 ± 2.5	39.1 ± 3.3	37.8 ± 2.3	sham vs. Stx+EPO: P = 0.005
	erythrocytes (cells/µl)	8162000 ± 337817	9196667 ± 477521	9200000 ± 811640	8926000 ± 546150	sham vs. Stx+vehicle: P = 0.0261
	Hb (g/dl)	11.3 ± 0.4	12.5 ± 0.6	12.6 ± 1	12.1 ± 0.8	sham vs. Stx+vehicle: P = 0.0374 sham vs. Stx+EPO: P = 0.0374
	thrombocytes (cells/µl)	890600 ± 71853	1078833 ± 123106	858167 ± 144950	1026400 ± 113372	Stx+vehicle vs. Stx+EPO: P = 0.0279
	leukocytes (cells/µl)	2500 ± 686	833.3 ± 151	1617 ± 799	1700 ± 957	sham vs. Stx+vehicle: P = 0.0059
	hemolysis score	0 ± 0	1.6 ± 1.4	1.3 ± 1.3	0.8 ± 1.1	sham vs. Stx+vehicle: P = 0.0023 sham vs. Stx+EPO: P = 0.013
**laboratory markers**	plasma ALAT (µmol/l*s)	0.6 ± 0.1	0.7 ± 0.2	0.6 ± 0.2	0.6 ± 0.2	ns
	plasma ASAT (µmol/l*s)	1.8 ± 1.1	2.6 ± 1.6	1.9 ± 1.1	1.3 ± 0.3	ns
	plasma LDH (µmol/l*s)	7.4 ± 2.9	16.4 ± 8.3	10.4 ± 4.6	8.1 ± 2.5	sham vs. Stx+vehicle: P = 0.0412
	plasma bilirubin (µmol/l)	2.0 ± 1.1	4.5 ± 1.5	5.0 ± 0.0	4.2 ± 1.1	sham vs. Stx+vehicle: P = 0.0153 sham vs. Stx+EPO: P = 0.0222
	plasma albumin (mg/dl)	13.5 ± 0.6	14.4 ± 1.0	15.0 ± 1.2	15.0 ± 0.7	sham vs. Stx+pHBSP: P = 0.0386

Hct, hematocrit; Hb, hemoglobin; ALAT, alanine aminotransferase; ASAT, aspartate aminotransferase; LDH, lactate dehydrogenase.

### Effect of EPO and pHBSP treatment on kidney injury and dysfunction in mice with HUS

**Figure 4:**
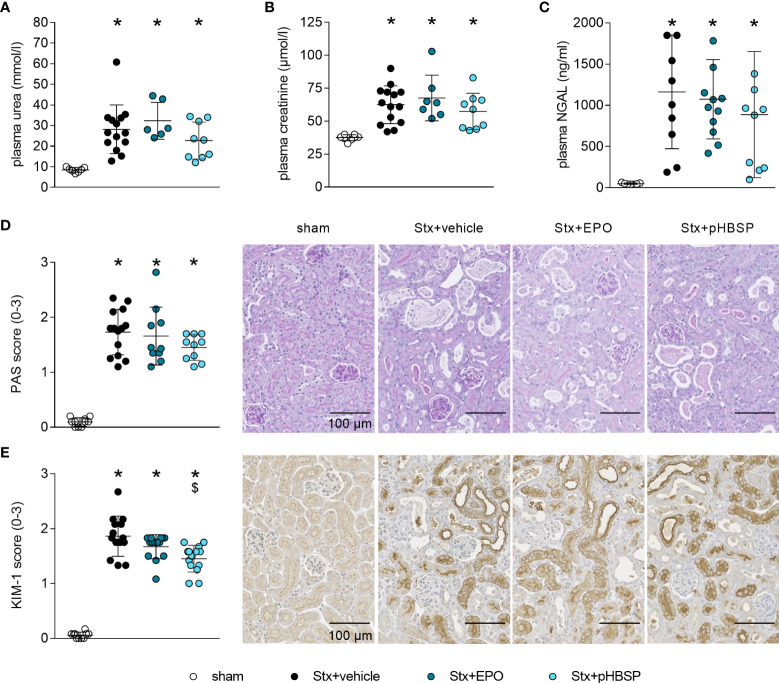
Effect of EPO and pHBSP treatment on kidney dysfunction and tubular injury in mice with HUS. Kidney injury and dysfunction was assessed on day 7 of HUS experiment. Plasma samples of mice with HUS and sham mice were analyzed for the kidney dysfunction parameters **(A)** urea and **(B)** creatinine (sham n = 7, Stx+vehicle n = 14, Stx+EPO n = 6, Stx+pHBSP n = 9) by an Architect™ ci16200 System (Abbott), as well as the kidney injury marker **(C)** NGAL (sham n = 7, Stx+vehicle n = 10, Stx+EPO n = 12, Stx+pHBSP n = 10) by ELISA. **(A-C)** Data are expressed as scatter dot plot with mean ± SD. *P < 0.05 vs. sham (one-way ANOVA, Holm-Sidak’s multiple comparison test). Quantification data as well as representative images (scale bar 100 µm) of **(D)** PAS staining (sham n = 10, Stx+vehicle n = 14, Stx+EPO n = 10, Stx+pHBSP n = 10, 10 fields per slide, score 0: no damage, 1: < 25% damaged, 2: 25–50% damaged, 3: > 50% damaged) and **(E)** immunohistochemical KIM-1 staining (score 0: < 25%, 1: 25–50%, 2: 50–75%, 3: > 75% strong positive staining per visual field, sham n = 10, Stx+vehicle n = 14, Stx+EPO n = 14, Stx+pHBSP n = 14, 12 fields per slide) in renal sections of sham and Stx-challenged mice. **(D, E)** Data are expressed as scatter dot plot with mean ± SD. *P < 0.05 vs. sham, ^$^P < 0.05 vs. Stx+vehicle, (Kruskal-Wallis test, Dunn’s multiple comparison test).

After observing significantly increased survival rates in EPO- and pHBSP-treated animals, we analyzed the impact of both treatments on kidney injury and dysfunction. Stx+vehicle, Stx+EPO and Stx+pHBSP mice presented with increased plasma urea, creatinine and NGAL compared with sham mice (**Figures 4A–C**). PAS staining revealed renal tissue damage in all Stx-challenged groups irrespective of treatment (**Figure 4D**). KIM-1 expression was elevated in Stx+vehicle, Stx+EPO and Stx+pHBSP mice compared with sham mice (**Figure 4E**). In Stx+pHBSP compared with Stx+vehicle mice, KIM-1 expression was reduced (**Figure 4E**).

### Effects of EPO and pHBSP treatment on intrarenal barriers in mice with HUS

**Figure 5:**
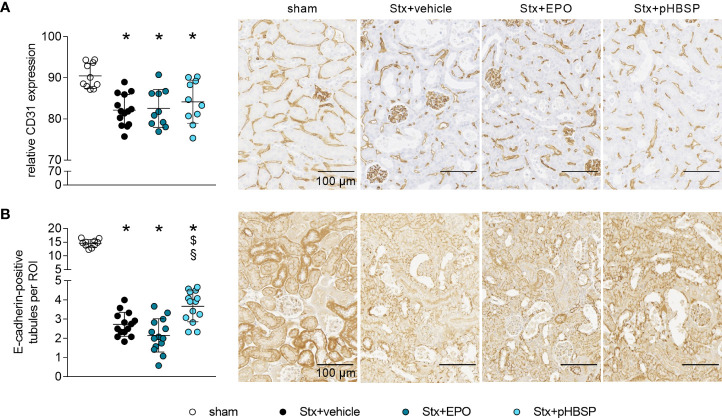
Effect of EPO and pHBSP treatment on intrarenal barriers in mice with HUS. Intrarenal barriers were assessed on day 7 of HUS experiment. Quantification data as well as representative images (scale bar 100 µm) of immunohistochemical **(A)** CD31 staining (sham n = 10, Stx+vehicle n = 14, Stx+EPO n = 10, Stx+pHBSP n = 10) and **(B)** E-cadherin (sham n = 10, Stx+vehicle n = 14, Stx+EPO n = 14, Stx+pHBSP n = 14), in renal sections of sham mice and mice subjected to Stx. **(A, B)** Data are expressed as scatter dot plot with mean ± SD. *P < 0.05 vs. sham, ^$^P < 0.05 vs. Stx+vehicle, ^§^P < 0.05 vs. Stx+EPO (one-way ANOVA, Holm-Sidak’s multiple comparison test).

The integrity of endothelial and epithelial barriers in the kidney is crucial for its function. It was demonstrated before that Stx can damage both endothelial as well as epithelial cells in the kidney and thereby influence the integrity of these barriers (1). We stained kidney sections for endothelial and epithelial cell markers to assess the influence of EPO and pHBSP treatment on intrarenal barriers. Loss of renal CD31-positive endothelial cells (**Figure 5A**) and reduced E-cadherin expression was detected in kidneys of all Stx-challenged groups compared with sham mice (**Figure 5B**). However, E-cadherin expression was less decreased in Stx+pHBSP compared with Stx+vehicle and Stx+EPO mice (**Figure 5B**).

### Effect of EPO and pHBSP treatment on microangiopathy in mice with HUS

**Figure 6:**
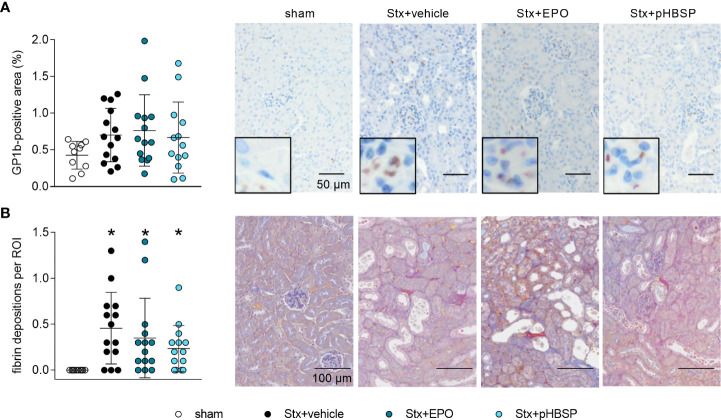
Effects of EPO and pHBSP treatment on microangiopathy in mice with HUS. Thrombocytes (indicated by glycoprotein 1b (GP1b) staining) and renal fibrin depositions (indicated by acid fuchsin orange G (SFOG) staining) were assessed on day 7 of HUS experiment. Quantification data and representative images of **(A)** immunohistochemical staining of thrombocytes (sham n = 10, Stx+vehicle n = 14, Stx+EPO n = 14, Stx+pHBSP n = 13) and **(B)** fibrin depositions (sham n = 10, Stx+vehicle n = 14, Stx+EPO n = 14, Stx+pHBSP n = 14) in kidney sections of sham mice and Stx-challenged mice. Quantitative data are expressed as scatter dot plot with mean ± SD. *P < 0.05 vs. sham (Kruskal-Wallis test, Dunn’s multiple comparison test), scale bar 100 µm.

GP1b was stained to highlight thrombocytes in the kidneys as indicators of microangiopathy (**Figure 6A**). An insignificant elevation of thrombocytes was observed in all Stx-challenged animals regardless of treatment, although it was weakest in the Stx+pHBSP group (**Figure 6A**). SFOG staining was performed to visualize fibrin deposits as indicators of microthrombi in renal sections (**Figure 6B**). Fibrin deposits were observed in all Stx-challenged groups compared with sham animals. There was an insignificant trend towards lower mean values of fibrin deposition in Stx+EPO and Stx+pHBSP mice compared with Stx+vehicle mice (**Figure 6B**).

### Effects of EPO and pHBSP treatment on nitrosative and oxidative stress in mice with HUS

**Figure 7:**
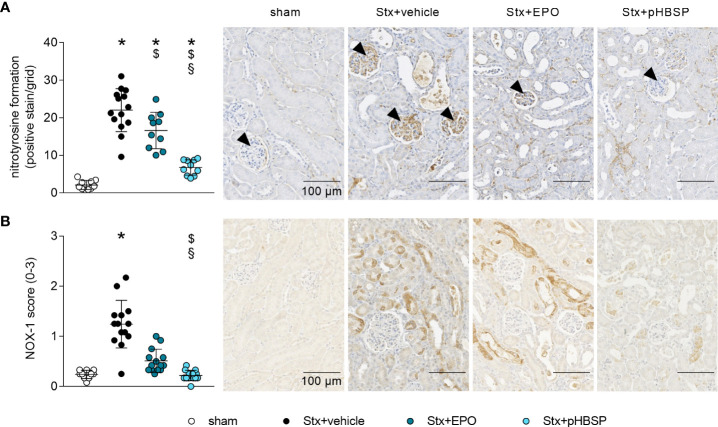
Effects of EPO and pHBSP treatment on nitrosative and oxidative stress in mice with HUS. Nitrosative and oxidative stress were assessed on day 7 of HUS experiment. Quantification data and representative images of immunohistochemical staining of **(A)** nitrotyrosine (sham n = 10, Stx+vehicle n = 14, Stx+EPO n = 10, Stx+pHBSP n = 10, arrowheads indicating glomeruli) and **(B)** NOX-1 (sham n = 10, Stx+vehicle n = 14, Stx+EPO n = 14, Stx+pHBSP n = 14) in kidney sections of sham mice and Stx-challenged mice. Quantitative data are expressed as scatter dot plot with mean ± SD. *P < 0.05 vs. sham, ^$^P < 0.05 vs. Stx+vehicle, ^§^P < 0.05 vs. Stx+EPO (A: one-way ANOVA, Holm-Sidak’s multiple comparison test, B: Kruskal-Wallis test, Dunn’s multiple comparison test), scale bar 100 µm.

To further characterize the potential protective mechanism of EPO and pHBSP in HUS, surrogate parameters of nitrosative (nitrotyrosin formation) and oxidative (NOX-1 expression) stress were analyzed. Renal nitrotyrosine formation was elevated in all Stx-challenged groups compared with sham mice (**Figure 7A**). Compared with Stx+vehicle mice, formation of nitrotyrosine was decreased in the kidneys of Stx+EPO and Stx+pHBSP mice (**Figure 7A**). Compared with Stx+EPO mice, nitrotyrosine staining was even further decreased in Stx+pHBSP mice (**Figure 7A**). Of note, the predominantly glomerular staining of nitrotyrosine in Stx+vehicle mice was mitigated in Stx+EPO mice and attenuated in Stx+pHBSP mice (**Figure 7A**, arrowheads). NOX-1 expression was increased only in Stx+vehicle compared with sham mice, whereas Stx+pHBSP mice displayed lower NOX-1 expression compared with Stx+vehicle and Stx+EPO mice (**Figure 7B**).

### Effect of EPO and pHBSP treatment on metabolome in mice with HUS

**Figure 8:**
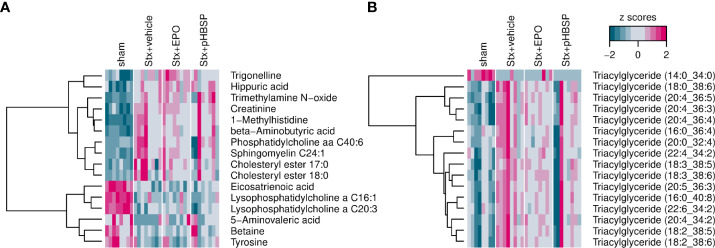
Effect of EPO and pHBSP treatment on plasma metabolome in mice with HUS. Metabolites in plasma of sham mice and Stx-challenged mice were assessed on day 7 of HUS experiment. Statistic modelling was performed to test the hypothesis that their abundance either increases or decreases in the following order: sham–Stx+pHBSP–Stx+EPO–Stx+vehicle **(A)** Small metabolites in murine plasma showing a significant trend for the four groups under investigation. **(B)** Triacylglyceroles in murine plasma showing a significant trend for the four groups under investigation. Data are shown as heatmaps depicting z-scores for all samples. The analytes were hierarchically clustered using Ward’s minimum variance method (45) and an euclidian distance between z scores. Dendograms provide information about distances between clusters.

Oxidative stress and metabolism are closely interrelated and studies on the metabolome have not yet been reported in animal models of HUS. Therefore, we performed targeted metabolomics in the plasma of all four groups. Of the 630 metabolites analyzed in plasma, 426 were within the limit of detection. These 426 metabolites were analyzed using a linear regression model to test whether their abundance increases or decreases in the assigned order: sham–Stx+pHBSP–Stx+EPO–Stx+vehicle. 32 metabolites fitted this hypothesis, they are highlighted in heatmaps (**Figures 8A, B**) and assigned to the following substance classes: amino acids and derivatives (7/32), alkaloids (1/32), aminoxides (1/32, heatmap A) and lipids (23/32, triacylglycerides in heatmap B).

## Discussion

To date, the role of EPO in HUS, a systemic orphan disease with occurrence of microangiopathic hemolytic anemia and AKI, has not been systematically investigated. We hypothesized that EPO treatment may be beneficial in patients with HUS-mediated hemolytic anemia and, targeting the IRR axis with EPO or non-hematopoietic EPO analogs, such as pHBSP, may convey nephroprotection in HUS.

### The role of endogenous EPO levels in patients, piglets and mice with STEC-HUS

In response to cellular hypoxia (46), a regulatory increase in renal EPO secretion in patients with anemia would be expected. To our knowledge, there are no systematic studies on changes in endogenous EPO levels in adult patients with STEC-HUS and only one brief report in pediatric patients (32). We observed increased EPO serum levels in a small cohort of adult patients with STEC-HUS compared to healthy controls already at a reduced Hb level of about 11 g/dl. Our data showed no correlation between the degree of anemia and EPO levels. This observation is consistent with the above-mentioned report in children with STEC-HUS (32) and in critically ill patients with AKI of other origins (47–50). However, we cannot exclude a gender bias, as our patient cohort was mainly female as women were more frequently affected in the 2011 EHEC outbreak (51). Therefore, our observations should be validated in a larger, gender balanced cohort.

In the kidneys, EPO is produced by fibroblast-like type I interstitial cells located between peritubular capillaries and the proximal convoluted tubule (52). Thus, EPO expression is likely to be impaired when EPO-producing cells are damaged. Consistent with these considerations, patients with very high LDH or creatinine levels as surrogate parameters for tissue damage and renal dysfunction had the lowest EPO levels in our study. However, we found no correlation between LDH or creatinine levels and EPO levels in patients with HUS. We attribute this to the small number of patients studied. Nevertheless, clinical data suggests that EPO response appears to be impaired in STEC-HUS and EPO regulation may play an important role in the pathogenesis and disease resolution. Analyzing serum EPO levels in a larger cohort of patients with HUS and acute kidney injury of other origins may help to further elucidate whether the degree of renal dysfunction and/or injury correlates with the endogenous EPO production. Here, we pursued our line of thought by further conducting studies in animals with experimental HUS.

In gnotobiotic piglets with EHEC infection (9) and in mice after Stx challenge, we found an increase in EPO expression despite an absence of anemia in these animal models. We and others reported earlier that the extent of systemic hemolysis and subsequent anemia in experimental HUS is not as pronounced as in patients with HUS (9, 10, 14, 53, 54).

Based on pathophysiological considerations and evidence, these findings could imply that EPO expression in HUS does not increase proportionally to the extent of anemia but rather depends on the degree of renal hypoxia as first mechanistically described by Semenza et al. (55, 56). Microthrombotic occlusion in the kidneys can result in local hypoxia increasing the expression of the master regulator hypoxia-induced factor 1α (HIF-1α) and thereby EPO (57). However, direct damage to the EPO-expressing cells in the kidneys of HUS patients with hemolytic anemia may, especially in cases with severe renal injury, also result in insufficient EPO production to adequately stimulate hematopoiesis. Thus, patients with severe renal injury could benefit from treatment with exogenous EPO (58, 59).

### EPO and pHBSP in the context of anemia correction and tissue protection

In our experimental setting, we observed a survival benefit in mice with HUS treated with either EPO or pHBSP. Tissue-protective effects of EPO and non-hematopoietic analogs have been the subject of discussion in the literature for nearly two decades (15, 16). Despite the observed beneficial effects of EPO in preclinical models of endotoxemia, sepsis, hemorrhagic shock and ischemia-reperfusion induced AKI (60–63), EPO failed to convey nephroprotection in several clinical trials that included patients following cardiac surgery (64, 65), cardiac arrest (66), kidney-transplantation (67) as well as ICU patients at risk for the development of AKI (EARLYARF trial) (68). However, in a small pilot trial with patients suffering from AKI after coronary artery bypass grafting surgery, treatment with EPO had a beneficial effect on all-cause mortality (69). Corwin et al. reported that administration of high-dose EPO (40,000 IU) to intensive care patients reduced the number of red blood cell transfusions without affecting mortality or clinical outcome (27). In a follow-up study of ICU patients, a subcohort of trauma patients treated with high-dose EPO showed no reduced need for red blood cell transfusions, however mortality was significantly reduced (18). Notably, an increase in thrombotic vascular events was noted in ICU patients who had not received thromboprophylaxis at baseline (18).

In the subset of mice surviving up to day 7 of the HUS experiments, we further investigated surrogate parameters for renal dysfunction and injury as well as barrier integrity, microangiopathy, oxidative and nitrosative stress and metabolome in plasma and/or renal tissue samples. As we have observed previously that AKI is accompanied by electrolyte imbalances, we propose that, in this model, mice die due to a severe AKI-induced hyperkalemia with subsequent cardiac arrhythmias and cardiac arrest (unpublished data). As fewer animals survived up to day 7 in the Stx+vehicle group (11/26, 42.3% survival) compared with the Stx+EPO (15/22, 68.2% survival) and Stx+pHBSP (16/21, 76.2% survival) group, the results need to be carefully interpreted in the light of a reverse survivorship bias, that might explain why we did not observe an effect of treatment with EPO or pHBSP on plasma creatinine, urea and NGAL, as surrogate parameters for AKI and renal dysfunction. However, we observed significant effects of EPO and/or pHBSP treatment on barrier integrity, oxidative and nitrosative stress and on selected metabolites in the samples studied.

Intact renal endothelial and epithelial barriers are important for the physiological function of the kidneys. We observed a pronounced Stx-induced damage of renal endothelial cells independent of treatment. Treatment with pHBSP, but not EPO led to a decrease in KIM-1 and an increase in E-cadherin expression in Stx-challenged mice in our setting. This could indicate less renal damage and enhanced epithelial barrier integrity, as tubular dedifferentiation – an important step in AKI progression – is characterised by a downregulation of E-cadherin (70). Although not resulting in amelioration of AKI in our model, this could have contributed to the increased survival observed after pHBSP treatment in Stx-challenged mice. In line with our results, the pharmaceutical upregulation of E-cadherin expression also proved nephroprotective in preclinical studies of cis-platin-induced AKI (71, 72).

In our recent study, we observed survival benefits in animals with reduced microangiopathy (11). Microangiopathy is a pathophysiological hallmark of HUS. As EPO can exert pro-thrombogenic effects, we consequently assessed the effects of EPO or pHBSP treatment on fibrin deposition and thrombocytes as surrogate parameters of microangiopathy to evaluate the potential of adverse treatment effects in HUS. Notably, we did not observe an aggravation of microthrombi formation or fibrin deposition in mice with HUS treated with either EPO or pHBSP.

Oxidative stress is involved in the pathogenesis of STEC-HUS (3–5). Nitrosative stress resembles a subtype of oxidative stress that has been shown to contribute to cell death (73). We observed an intense glomerular staining of nitrotyrosine in Stx+vehicle mice. Nitrotyrosine results from the reaction of the peroxynitrite radical – created when excess nitric oxide (NO) and superoxide radicals combine (74) – with accessible tyrosine residues (75). While basal NO plays a critical role in the regulation of the perfusion and vascular tone in glomeruli (74), it has been shown previously that inducible NO synthase (iNOS) induction and overproduction of NO had deleterious effects in ischemic AKI (76). In mice, iNOS is – among others – expressed in macrophages and glomerular mesangial cells and inducible by various pro-inflammatory stimuli (77). EPO and pHBSP have been demonstrated to reduce the expression of iNOS either on protein level in AKI of different etiologies (63, 78) or on mRNA level in lung and brain injury (79, 80). Thus, the reduction in renal nitrotyrosine staining in Stx+EPO and Stx+pHBSP mice could result from an inhibition of iNOS in the glomeruli of these mice.

Due to its critical role in the reabsorption of nutrients and maintenance of homeostasis, energy demand and metabolic activity are high in the kidneys (81). By analyzing the plasma metabolome, we observed an increase in triglycerides and cholesteryl esters in Stx-challenged mice that was consistently reported in HUS patients (82). Notably, we observed that alterations in some metabolites associated with kidney injury, e. g. trimethylamine N-oxide (83, 84) and trigonelline (85, 86) as well as lipid compounds were less distinct in mice with HUS after EPO or pHBSP treatment. In line with our results, a recent study demonstrated that EPO reduces lipidemia by stimulating lipid catabolism in peripheral adipose tissue (87). Further studies are required to examine the complex metabolic changes in animals and patients with HUS.

Taken together, we found that treatment with EPO or pHBSP positively affects several pathomechanisms of STEC-HUS which might explain the observed improvement in clinical outcome of mice with HUS. Further systematic studies in this context in animal models and even more importantly larger patient cohorts are needed to provide sufficient evidence to adjust clinical management.

## Conclusion

We report here for the first time, that I) EPO levels in patients with STEC-HUS, STEC-infected piglets and Stx-challenged mice are elevated in a uniform manner, II) EPO or pHBSP treatment of mice with HUS improves survival and disease outcome, III) protective effects of pHBSP and EPO are associated with reduced renal oxidative stress, and IV) treatment with pHBSP in Stx-challenged mice is additionally associated with ameliorated nitrosative stress, less KIM-1 expression and tubular dedifferentiation. In the light of our results demonstrating favourable tissue-protective effects of EPO in a preclinical model, treatment of HUS-induced hemolytic anemia with EPO should be considered in patients. Further studies are needed to evaluate the effect of EPO and pHBSP treatment in clinical studies in patients with STEC-HUS.

## Data availability statement

The metabolomics dataset presented in this study can be found in online repositories. The names of the repository/repositories and accession number(s) can be found below: https://www.ebi.ac.uk/biostudies/BioStudies accession number S-BSST657. Original data can be requested from sina.coldewey@med.uni-jena.de.

## Ethics statement

The studies involving human participants were reviewed and approved by Ethics Committee of Hannover Medical School (1123-2011) and Ethics Committee of Friedrich Schiller University Jena (5276-09/17). The patients/participants provided their written informed consent to participate in this study. The animal studies were reviewed and approved by the Lower Saxony State Office for Consumer Protection and Food Safety (Approval Number: 33.9-42502-04-13/1149) and the regional animal welfare committee and the Thuringian State Office for Consumer Protection (registration number 02-058/14).

## Author contributions

SC designed and planned the study; planned, performed and analyzed experiments and wrote the manuscript. SD planned, performed and analyzed experiments and wrote the manuscript. WP planned and performed histology and immunohistochemistry and analyzed data. BW planned and performed animal experiments. FG provided purified Stx and gnotobiotic piglet samples and contributed important intellectual content. DI synthesized and purified pHBSP and contributed important intellectual content. CD and KA performed histology and immunohistochemistry and contributed important intellectual content to histology and immunohistochemistry. JK provided HUS patient samples. IH-P provided gnotobiotic piglet samples. All authors provided important intellectual content and revised the manuscript prior to submission.

## Funding

The research leading to these results has received funding from the German Research Foundation (DFG; Research Unit FOR1738, grant no. CO912/2-1 to SC and IM97/7-2 to DI) and the Federal Ministry of Education and Research (BMBF; ZIK Septomics Research Center, Translational Septomics, grant no. 03Z22JN12 to SC and Center for Sepsis Control and Care, project TaSep, grant no. 01EO1502 to SC).

## Acknowledgments

We would like to thank J. Fischer (Septomics Research Center, Jena) and Dr. H. H. Brewitz (University of Bonn) for technical assistance, Prof. Dr. J. Menne (KRH Klinikum Siloah, Hannover) for provision of HUS patient samples and D. Driesch (BioControl) for support in metabolomics data analysis.

## Conflict of interest

The authors declare that the research was conducted in the absence of any commercial or financial relationships that could be construed as a potential conflict of interest.

## Publisher’s note

All claims expressed in this article are solely those of the authors and do not necessarily represent those of their affiliated organizations, or those of the publisher, the editors and the reviewers. Any product that may be evaluated in this article, or claim that may be made by its manufacturer, is not guaranteed or endorsed by the publisher.
