# Supplementary material for: Targeting the innate repair receptor axis via erythropoietin or pyroglutamate helix B surface peptide attenuates hemolytic-uremic syndrome in mice
Source: Front Immunol. 2022 Sep 23;13:1010882. doi: 10.3389/fimmu.2022.1010882 (PMC9537456; doi:10.3389/fimmu.2022.1010882)
Supplement: Supplementary file 1 [file DataSheet_1.docx]

Supplementary Material

**Targeting the innate repair receptor axis
via erythropoietin or pyroglutamate helix B surface peptide attenuates hemolytic-uremic syndrome in mice**

**Authors: Sophie Dennhardt^1,2,3^, Wiebke Pirschel^1,2^, Bianka Wissuwa^1,2^, Diana Imhof^4^, Christoph Daniel^5^, Jan T. Kielstein^6^, Isabel Hennig-Pauka^7^, Kerstin Amann^5^_,_ Florian Gunzer^8^, Sina M. Coldewey^1,2,3*^**

^1^Department of Anesthesiology and Intensive Care Medicine, Jena University Hospital; Jena, Germany.

^2^Septomics Research Center, Jena University Hospital; Jena, Germany.

^3^Center for Sepsis Control and Care, Jena University Hospital; Jena, Germany.

^4^Pharmaceutical Biochemistry and Bioanalytics, Pharmaceutical Institute, University of Bonn; Bonn, Germany.

^5^Department of Nephropathology, Friedrich-Alexander University (FAU) Erlangen-Nürnberg; Erlangen, Germany.

^6^Medical Clinic V, Nephrology | Rheumatology | Blood Purification, Academic Teaching Hospital Braunschweig; Braunschweig, Germany.

^7^Field Station for Epidemiology, University of Veterinary Medicine Hannover; Bakum, Germany.

^8^Department of Hospital Infection Control, University Hospital Carl Gustav Carus, TU Dresden; Dresden, Germany.

***Correspondence:**

Prof. Sina M. Coldewey, MD, PhD,

sina.coldewey@med.uni-jena.de

# Supplementary Methods

# *pHBSP peptide synthesis*

# All chemicals for the peptide synthesis of pHBSP were purchased from Iris Biotech GmbH, Orpegen Peptide Chemicals GmbH or Merck Millipore. The peptide ZEQLERALNSS-OH (Z: Pyr) was synthesized according to a standard Fmoc (N-(9-fluorenyl)methoxycarbonyl) protocol for automated solid-phase peptide synthesis employing an EPS 221 peptide synthesizer (Intavis Bioanalytical Instruments AG). A Ser(tBu)-preloaded chlorotrityl chloride resin (120 mg, 1.00 mmol/g) was used for subsequent peptide elongation with HBTU (4 equiv.) as coupling reagent and NMM (8 equiv.) as the base. Peptide cleavage was performed at room temperature for 3 h in reagent K cleavage mixture (75 mg phenol, 25 µl ethanditiol, 50 µl thioanisol, 50 µl water, 950 µl trifluoroacetic acid per 100 mg resin). The cleavage solution was filtered and the peptide precipitated in cold diethyl ether and washed three times with diethyl ether. The crude peptide was purified by semipreparative reversed-phase HPLC using a Shimadzu LC-8A system equipped with a C18 column (Knauer Eurospher 100, 5 µm particle size, 100 Å pore size, 250 x 32 mm). Solvents for gradient elution were 0.1% TFA in water (eluent A) and 0.1% TFA in acetonitrile/water (90:10, eluent B). Detection was performed at 220 nm. Purity of the peptide was confirmed by analytical reversed-phase HPLC on a Shimadzu LC-10AT chromatograph equipped with a Vydac 218TP column (250 x 4.6 mm, 5 µm particle size, 300 Å pore size). pHBSP was analyzed using a gradient from 0% to 60% eluent B (0.1% TFA in acetonitrile) in eluent A (0.1% TFA in water) for 60 min. A retention time of 36.1 min was observed for pure pHBSP (purity HPLC > 98%). Identity of PHBSP was confirmed by LC-ESI-MS analysis on a micrOTOF-Q III system (Bruker Daltonics GmbH) connected to a Dionex Ultimate 3000 (Thermo Scientific). Samples were separated by an EC 100/2 Nucleoshell RP18 column (C18 reversed phase, 100 x 2 mm, 2.7 µm particle size, 90 Å pore size). The correct molar mass (m/z 1257.61 g/mol [M+H]^+^) was detected (theoretical mass m.i. 1256.60 g/mol). Amino acid analysis using an Eppendorf Amino Acid Analyser LC 3000 (Eppendorf) after hydrolysis with 6 N HCl at 110 °C for 24 h revealed the expected amino acid content according to the primary sequence.

# Supplementary Figures


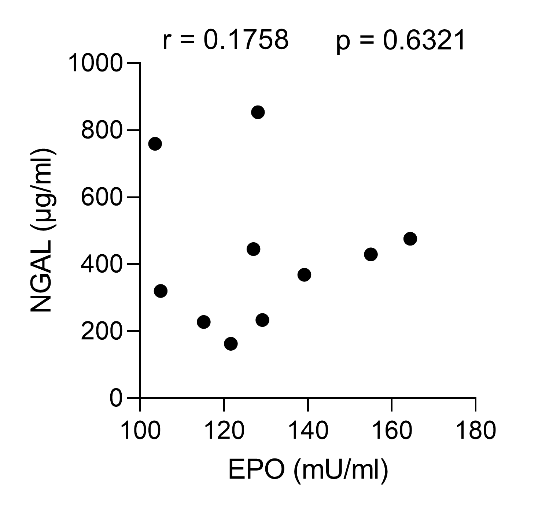


Supplementary Figure 1: Spearman’s correlation for murine plasma EPO and NGAL. Correlation coefficient (r) and P value are given for n = 10 mice.

# Supplementary Tables

# Table S1: ELISA kits used for analysis in serum (EPO porcine) or plasma (EPO human and murine, NGAL).

| **parameter** | **supplier** | **sample dilution** | **LOT-no.** | **cat. no.** |
| --- | --- | --- | --- | --- |
| EPO (human) | abcam | 1:2 | GR3249711-1 | ab119522 |
| EPO (murine) | Cloud-Clone Corp. | 1:16 | L141101096 | SEA028Mu |
| EPO (porcine) | Novateinbio | - | 121002KB | BG-POR10827 |
| NGAL | BioLegend | sham: 1:200  Stx: 1:1,000 | B228519 | 443708 |

**Table S2: Primary antibodies and their dilutions used for immunohistochemical staining.**

| **antibody** | **supplier** | **dilution** | **batch** | **cat. no.** |
| --- | --- | --- | --- | --- |
| polyclonal goat anti-KIM-1 | R&D Systems | 1:1000 in TRIS buffer + 1% BSA | KCA0415031 | AF1817 |
| monoclonal rat anti-CD31 | Dianova | 1:100 in TRIS buffer + 1% BSA | 15219/01 | DIA-310 |
| monoclonal mouse anti-E-cadherin | BD Transduction Laboratories^TM^ | 1:200 in prediluted diluent (M.O.M. kit) | 66000 | 610181 |
| polyclonal rabbit anti-NOX-1 | Antibodies-online | 1:1000 in TRIS buffer | 0161212c026631 | PA1666 |
| polyclonal rabbit anti-nitrotyrosine | Upstate Biotechnology | 1:200 in TRIS buffer + 1% BSA | 32601 | 06-284 |
| anti-glycoprotein 1b alpha | Emfret Analytics GmbH & Co. KG | 1:200 in TRIS buffer | Clone Xia.G5 | M040-0 |

**Table S3: Secondary antibodies and their dilutions used for immunohistochemical staining.**

| **antibody** | **supplier** | **dilution** | **batch** | **cat. no.** |
| --- | --- | --- | --- | --- |
| biotinylated anti-rabbit IgG (H+L) | Vector Laboratories | 1:200 in TRIS buffer | ZE0730 | BA-1000 |
| biotinylated anti-goat IgG (H+L) | Vector Laboratories | 1:500 in TRIS buffer | ZA0425 | BA-5000 |
| Biotinylated anti-rat IgG (H+L) | Vector Laboratories | 1:200 in TRIS buffer  + 1% BSA | ZE0201 | BA-4001 |

Table S4: Statistical analysis (mean±SD and P-values) for all parameters analyzed in the manuscript.

| parameter | group | mean±SD | P-values | | | |
| --- | --- | --- | --- | --- | --- | --- |
|  |  |  | vs. healthy control | vs. STEC-HUS T1 | vs. STEC HUS T2 |  |
| EPO (Fig. 1A) | healthy control | 5.11±4.254 | - | <0.0001 | 0.0068 |  |
|  | STEC-HUS T1 | 31.19±34.26 | <0.0001 | - | 0.0024 |  |
|  | STEC-HUS T2 | 15.02±17.27 | 0.0068 | 0.0024 | - |  |
|  | | | vs. sham | vs. EHEC O104:H11 | vs. EHEC O157:H7 |  |
| EPO (Fig. 2A) | sham | 18.34±3.202 | - | 0.0447 | 0.0010 |  |
|  | EHEC O104:H4 | 23.38±3.998 | 0.0447 | - | 0.0510 |  |
|  | EHEC O157:H7 | 28.32±3.207 | 0.0010 | 0.0510 | - |  |
|  | | | vs. sham | vs. Stx |  | |
| EPO (Fig. 2B) | sham | 107.4±19.09 | - | 0.0446 |  | |
|  | Stx | 128.8±20.95 | 0.0446 | - |  |  |
|  | | | vs. sham | vs. Stx+vehicle | vs. Stx+EPO | vs.Stx+pHBSP |
| survival (Fig. 3A) | sham | - | - | 0.0004 | - | - |
|  | Stx+vehicle | - | 0.0004 | - | 0.0290 | 0.0141 |
|  | Stx+EPO | - | - | 0.0290 | - | - |
|  | Stx+pHBSP | - | - | 0.0141 | - | - |
| weight loss day 1 (Fig. 3B) | sham | 0.320±2.573 | - | >0.9999 | >0.9999 | >0.9999 |
|  | Stx+vehicle | -1.019±2.275 | >0.9999 | - | >0.9999 | >0.9999 |
|  | Stx+EPO | -0.584±2.139 | >0.9999 | >0.9999 | - | >0.9999 |
|  | Stx+pHBSP | -1.045±1.722 | >0.9999 | >0.9999 | >0.9999 | - |
| weight loss day 2 (Fig. 3B) | sham | 0.344±2.692 | - | 0.1201 | >0.9999 | 0.4152 |
|  | Stx+vehicle | -2.445±2.491 | 0.1201 | - | >0.9999 | >0.9999 |
|  | Stx+EPO | -1.122±3.099 | >0.9999 | >0.9999 | - | >0.9999 |
|  | Stx+pHBSP | -1.926±2.774 | 0.4152 | >0.9999 | >0.9999 | - |
| weight loss day 3 (Fig. 3B) | sham | 0.365±2.547 | - | 0.0005 | 0.2271 | 0.0070 |
|  | Stx+vehicle | -4.392±3.377 | 0.0005 | - | 0.2470 | >0.9999 |
|  | Stx+EPO | -2.205±3.344 | 0.2271 | 0.2470 | - | >0.9999 |
|  | Stx+pHBSP | -3.705±3.624 | 0.0070 | >0.9999 | >0.9999 | - |
| weight loss day 4 (Fig. 3B) | sham | -1.683±2.451 | - | <0.0001 | 0.0001 | <0.0001 |
|  | Stx+vehicle | -10.366±4.459 | <0.0001 | - | 0.0117 | >0.9999 |
|  | Stx+EPO | -7.040±3.067 | 0.0001 | 0.0117 | - | 0.3484 |
|  | Stx+pHBSP | -9.177±4.357 | <0.0001 | >0.9999 | 0.3484 | - |
| weight loss day 5 (Fig. 3B) | sham | -2.222±1.696 | - | <0.0001 | <0.0001 | <0.0001 |
|  | Stx+vehicle | -15.381±4.787 | <0.0001 | - | 0.0049 | 0.7028 |
|  | Stx+EPO | -11.787±3.641 | <0.0001 | 0.0049 | - | 0.5550 |
|  | Stx+pHBSP | -13.683±4.628 | <0.0001 | 0.7028 | 0.5550 | - |
| HUS score 0 h (Fig. 3C) | sham | 1±0 | - | - | - | - |
|  | Stx+vehicle | 1±0 | - | - | - | - |
|  | Stx+EPO | 1±0 | - | - | - | - |
|  | Stx+pHBSP | 1±0 | - | - | - | - |
| HUS score 24 h (Fig. 3C) | sham | 1±0 | - | - | - | - |
|  | Stx+vehicle | 1±0 | - | - | - | - |
|  | Stx+EPO | 1±0 | - | - | - | - |
|  | Stx+pHBSP | 1±0 | - | - | - | - |
| HUS score 48 h (Fig. 3C) | sham | 1±0 | - | >0.9999 | 0.8753 | >0.9999 |
|  | Stx+vehicle | 1.038±0.196 | >0.9999 | - | >0.9999 | >0.9999 |
|  | Stx+EPO | 1.091±0.294 | 0.8753 | >0.9999 | - | 0.6628 |
|  | Stx+pHBSP | 1±0 | >0.9999 | >0.9999 | 0.6628 | - |
| HUS score 72 h (Fig. 3C) | sham | 1±0 | - | 0.0083 | 0.1456 | 0.0512 |
|  | Stx+vehicle | 1.5±0.510 | 0.0083 | - | >0.9999 | >0.9999 |
|  | Stx+EPO | 1.364±0.492 | 0.1456 | >0.9999 | - | >0.9999 |
|  | Stx+pHBSP | 1.429±0.507 | 0.0512 | >0.9999 | >0.9999 | - |
| HUS score 96 h (Fig. 3C) | sham | 1±0 | - | <0.0001 | <0.0001 | <0.0001 |
|  | Stx+vehicle | 1.885±0.432 | <0.0001 | - | >0.9999 | >0.9999 |
|  | Stx+EPO | 1.818±0.395 | <0.0001 | >0.9999 | - | >0.9999 |
|  | Stx+pHBSP | 1.905±0.301 | <0.0001 | >0.9999 | >0.9999 | - |
| HUS score 120 h (Fig. 3C) | sham | 1±0 | - | <0.0001 | <0.0001 | <0.0001 |
|  | Stx+vehicle | 2.808±1.327 | <0.0001 | - | 0.2644 | 0.7638 |
|  | Stx+EPO | 2.045±0.213 | <0.0001 | 0.2644 | - | >0.9999 |
|  | Stx+pHBSP | 2.143±0.478 | <0.0001 | 0.7638 | >0.9999 | - |
| HUS score 144 h (Fig. 3C) | sham | 1±0 | - | <0.0001 | <0.0001 | <0.0001 |
|  | Stx+vehicle | 4.115±2.179 | <0.0001 | - | 0.6948 | 0.2570 |
|  | Stx+EPO | 2.818±1.22 | <0.0001 | 0.6948 | - | >0.9999 |
|  | Stx+pHBSP | 2.667±1.39 | <0.0001 | 0.2570 | >0.9999 | - |
| HUS score 168 h (Fig. 3C) | sham | 1±0 | - | <0.0001 | <0.0001 | <0.0001 |
|  | Stx+vehicle | 5.192±2.298 | <0.0001 | - | 0.9507 | >0.9999 |
|  | Stx+EPO | 4.091±2.328 | <0.0001 | 0.9507 | - | >0.9999 |
|  | Stx+pHBSP | 4.048±2.061 | <0.0001 | >0.9999 | >0.9999 | - |
| creatinine (Fig. 4A) | sham | 37.5±2.268 | - | 0.0008 | 0.0007 | 0.0159 |
|  | Stx+vehicle | 62.5±14.31 | 0.0008 | - | 0.6151 | 0.6151 |
|  | Stx+EPO | 67.57±17.34 | 0.0007 | 0.6151 | - | 0.3636 |
|  | Stx+pHBSP | 57.44±13.71 | 0.0159 | 0.6151 | 0.3636 | - |
| urea (Fig. 4B) | sham | 8.529±1.197 | - | 0.0005 | 0.0005 | 0.0216 |
|  | Stx+vehicle | 28.19±11.81 | 0.0005 | - | 0.3743 | 0.3375 |
|  | Stx+EPO | 32.35±8.954 | 0.0005 | 0.3743 | - | 0.1762 |
|  | Stx+pHBSP | 22.73±8.94 | 0.0216 | 0.3375 | 0.1762 | - |
| NGAL (Fig. 4C) | sham | 49.9±8.9 | - | 0.0029 | 0.0041 | 0.0261 |
|  | Stx+vehicle | 1163±689.9 | 0.0029 | - | 0.7250 | 0.6582 |
|  | Stx+EPO | 1074±481.2 | 0.0041 | 0.7250 | - | 0.7114 |
|  | Stx+pHBSP | 887.1±765.1 | 0.0261 | 0.6582 | 0.7114 | - |
| PAS (Fig. 4D) | sham | 0.095±0.08 | - | <0.0001 | 0.0007 | 0.0112 |
|  | Stx+vehicle | 1.732±0.41 | <0.0001 | - | >0.9999 | >0.9999 |
|  | Stx+EPO | 1.66±0.53 | 0.0007 | >0.9999 | - | >0.9999 |
|  | Stx+pHBSP | 1.45±0.23 | 0.0112 | >0.9999 | >0.9999 | - |
| KIM-1 (Fig. 4E) | sham | 0.057±0.056 | - | <0.0001 | <0.0001 | 0.0359 |
|  | Stx+vehicle | 1.863±0.366 | <0.0001 | - | >0.9999 | 0.0315 |
|  | Stx+EPO | 1.671±0.220 | <0.0001 | >0.9999 | - | 0.3643 |
|  | Stx+pHBSP | 1.451±0.244 | 0.0359 | 0.0315 | 0.3643 | - |
| CD31 (Fig. 5A) | sham | 90.48±3.031 | - | 0.0001 | 0.0007 | 0.0070 |
|  | Stx+vehicle | 82.16±3.799 | 0.0001 | - | 0.8174 | 0.5865 |
|  | Stx+EPO | 82.57±4.602 | 0.0007 | 0.8174 | - | 0.6378 |
|  | Stx+pHBSP | 84.17±5.222 | 0.0070 | 0.5865 | 0.6378 | - |
| E-cadherin (Fig. 5B) | sham | 14.62±1.394 | - | <0.0001 | <0.0001 | <0.0001 |
|  | Stx+vehicle | 2.731±0.621 | <0.0001 | - | 0.1036 | 0.0187 |
|  | Stx+EPO | 2.154±0.865 | <0.0001 | 0.1036 | - | 0.0002 |
|  | Stx+pHBSP | 3.672±0.805 | <0.0001 | 0.0187 | 0.0002 | - |
| GP1b (Fig. 6A) | sham | 0.427±0.190 | - | 0.4413 | 0.3588 | >0.9999 |
|  | Stx+vehicle | 0.702±0.364 | 0.4413 | - | >0.9999 | >0.9999 |
|  | Stx+EPO | 0.765±0.486 | 0.3588 | >0.9999 | - | >0.9999 |
|  | Stx+pHBSP | 0.669±0.484 | >0.9999 | >0.9999 | >0.9999 | - |
| SFOG (Fig. 6B) | sham | 0±0 | - | 0.0005 | 0.0096 | 0.0411 |
|  | Stx+vehicle | 0.457±0.390 | 0.0005 | - | >0.9999 | >0.9999 |
|  | Stx+EPO | 0.350±0.433 | 0.0096 | >0.9999 | - | >0.9999 |
|  | Stx+pHBSP | 0.236±0.250 | 0.0411 | >0.9999 | >0.9999 | - |
| nitrotyrosine (Fig. 7A) | sham | 2.105±1.205 | - | <0.0001 | <0.0001 | 0.0154 |
|  | Stx+vehicle | 22.04±5.708 | <0.0001 | - | 0.0060 | <0.0001 |
|  | Stx+EPO | 16.63±4.839 | <0.0001 | 0.0060 | - | <0.0001 |
|  | Stx+pHBSP | 6.79±2.038 | 0.0154 | <0.0001 | <0.0001 | - |
| NOX-1 (Fig. 7B) | sham | 0.241±0.082 | - | <0.0001 | 0.0746 | >0.9999 |
|  | Stx+vehicle | 1.244±0.475 | <0.0001 | - | 0.1424 | <0.0001 |
|  | Stx+EPO | 0.511±0.232 | 0.0746 | 0.1424 | - | 0.0133 |
|  | Stx+pHBSP | 0.219±0.104 | >0.9999 | <0.0001 | 0.0133 | - |

**Table S5: Characteristics of HUS patients analyzed in this study.**

| patient no. | sex | age | EPO (acute stage) in mU/ml | EPO (pre-discharge) in mU/ml |
| --- | --- | --- | --- | --- |
| 1 | female | 65 | 13.482 | 8.526 |
| 2 | female | 45 | 11.618 | 7.958 |
| 3 | female | 28 | 67.768 | 19.73 |
| 4 | female | 51 | 27.762 | 8.314 |
| 5 | male | 26 | 28.194 | 8.946 |
| 6 | female | 47 | 91.38 | 60.204 |
| 7 | male | 75 | 15.314 | 29.546 |
| 8 | female | 48 | 26.282 | 4.626 |
| 9 | female | 74 | 19.856 | 10.466 |
| 10 | female | 23 | 10.466 | 68.41 |
| 11 | female | 29 | 40.382 | 10.054 |
| 12 | female | 39 | 29.546 | 12.422 |
| 13 | female | 44 | 19.034 | 5.92 |
| 14 | female | 45 | 147.312 | 10.26 |
| 15 | female | 50 | 29.914 | 3.428 |
| 16 | female | 27 | 15.314 | 6.366 |
| 17 | male | 47 | 92.482 | 4.626 |
| 18 | female | 38 | 0 | 1.506 |
| 19 | female | 77 | 6.366 | 5.846 |
| 20 | female | 63 | 5.47 | 7.314 |
| 21 | female | 42 | 18.652 | 2.846 |
| 22 | male | 58 | 20.612 | 28.316 |
| 23 | female | 47 | 16.414 | 4.312 |
| 24 | female | 46 | 11.686 | 4.704 |
| 25 | female | 73 | 11.82 | 20.108 |
| 26 | female | 74 | 83.838 | 41.362 |
| 27 | female | 20 | 17.824 | 14.01 |

**Table S6: Characteristics of healthy controls analyzed in this study.**

| healthy control no. | sex | age | EPO in mU/ml |
| --- | --- | --- | --- |
| 1 | female | 39 | 2.588 |
| 2 | male | 60 | 1.694 |
| 3 | female | 63 | 7.674 |
| 4 | female | 65 | 10.67 |
| 5 | male | 65 | 5.696 |
| 6 | female | 67 | 10.466 |
| 7 | male | 74 | 1.97 |
| 8 | female | 65 | 5.166 |
| 9 | female | 50 | 0 |
| 10 | male | 52 | 5.92 |
| 11 | female | 40 | 0 |
| 12 | female | 53 | 4.704 |
| 13 | female | 67 | 8.736 |
| 14 | female | 62 | 6.144 |
| 15 | female | 56 | 1.6 |
| 16 | female | 57 | 0 |
| 17 | female | 71 | 7.098 |
| 18 | female | 64 | 0.584 |
| 19 | male | 68 | 16.028 |
| 20 | male | 72 | 5.47 |

Table S7: Spearman correlation coefficients between endogenous EPO levels and hemoglobin (Hb), hematocrit (Hct), lactate dehydrogenase (LDH) and creatinine (Crea) in mock-infected (sham), O104:H4- and O157:H7-infected gnotobiotic piglets 4-6 days after infection.

| Spearman correlation | | | | | | | | |
| --- | --- | --- | --- | --- | --- | --- | --- | --- |
|  | correlation coefficients (r) and *P*-values (p) | | | | | | | |
|  | Hb | | Hct | | LDH | | Crea | |
|  | r | p | r | p | r | p | r | p |
| sham | 0.05 | 0.9349 | -0.31 | 0.4501 | 0.29 | 0.5008 | 0.83 | 0.0154 |
| O104:H4 | 0.3 | 0.6833 | 0.1 | 0.9500 | 0.7 | 0.2333 | 0.5 | 0.4500 |
| O157:H7 | -0.5 | >0.9999 | -0.5 | >0.9999 | -1 | 0.0833 | 0.4 | 0.7500 |
